# Diverse Expression of IL-32 in Diffuse and Intestinal Types of Gastric Cancer

**DOI:** 10.1155/2018/6578273

**Published:** 2018-10-04

**Authors:** Mladen Pavlovic, Nevena Gajovic, Milena Jurisevic, Slobodanka Mitrovic, Gordana Radosavljevic, Jelena Pantic, Nebojsa Arsenijevic, Ivan Jovanovic

**Affiliations:** ^1^Department of Surgery, Faculty of Medical Sciences, University of Kragujevac, Serbia; ^2^Center for Molecular Medicine and Stem Cell Research, Faculty of Medical Sciences, University of Kragujevac, Serbia; ^3^Department of Pharmacy, Faculty of Medical Sciences, University of Kragujevac, Serbia; ^4^Department of Pathology, Faculty of Medical Sciences, University of Kragujevac, Serbia

## Abstract

**Introduction:**

Gastric cancer (GC) represents one of the most common cancers worldwide, frequently diagnosed at advanced stages with poor prognosis, indicating on need for new diagnostic and prognostic markers. The aim of the study was to determine the expression of IL-32, proinflammatory and angiogenic mediators, in patients with diffuse and intestinal gastric cancer and the relationship with clinicopathological aspects.

**Material and Methods:**

The tissue samples of diffuse and intestinal types of tumor of 70 patients with gastric cancer were analyzed. Expression of IL-32, VEGF, IL-17, and CD31 was measured by immunohistochemistry.

**Results:**

IL-32 expression was significantly lower in tissue samples from patients with diffuse type of gastric cancer that is also a severe and more progressive form (TNM stages III and IV, poor histological differentiation, and higher nuclear grade III). Expression of IL-17 was also decreased in patients with diffuse type of gastric cancer. Microvascular density was diminished in diffuse type of gastric cancer.

**Conclusions:**

Downregulated expression of IL-32 in tumor tissue of patients with diffuse type of gastric cancer may implicate on its role in limiting ongoing proinflammatory and proangiogenic processes. This emphasizes on unrecognized role of IL-32 in biology of diffuse type of gastric cancer.

## 1. Introduction

Gastric cancer is the fourth most common type of cancer and the second cause of cancer-related deaths after lung cancer [1, 2]. The various incidence of gastric cancer among population is considered to be mainly associated with variations in diet [3, 4]. The poor prognosis of this type of tumor is mainly because of late diagnosis and because the early stages do not give any clinical manifestations.

One of the most widely used histological classification of gastric cancer is based on Lauren's criteria, in which gastric adenocarcinoma is a heterogeneous disease histologically divided into intestinal, diffuse, mixed, and indeterminate subtypes [4, 5] and can be anatomically classified as proximal or distal type of tumor [2, 6]. These two types of tumors differ in morphology, epidemiology, progression pattern, genetic basis, and clinical manifestations. Intestinal tumor cells are often adhesive metastatic cells that usually form tubular or glandular structures [1, 7, 8]. Intestinal type of gastric cancer spreads via lymphatic or vascular vessels, and the lesions are irregularly straggled. Diffuse gastric cancer consists of nonadhesive cells that predominantly infiltrate stroma, that is not characteristic of intestinal form. The fact that diffuse type of gastric cancer mainly invades peritoneum cavity is one of the main reasons for shorter duration of disease and poor prognosis [7, 9, 10].

Inflammation and angiogenesis are important factors for carcinogenesis that have big impact on progression and invasion of tumor cells [11–13]. Numerous investigations have revealed various molecular and cellular pathways that are vital for linking inflammation and cancer [13–15]. The effect of immune cells on tumor cells partly depends on the production of cytokines, chemokines, growth factors, and reactive oxygen species [16].

One of the most intriguing among numerous cytokines that has role in both hallmarks of cancer is recently described interleukin 32. IL-32 induces the production of proinflammatory cytokines and also directly affects the development and maturation of specific immune cells [17, 18]. IL-32 is also involved in numerous inflammatory and infectious diseases, including rheumatoid arthritis, chronic obstructive pulmonary disease, mycobacterium tuberculosis infections, and inflammatory bowel disease [19–23]. Regarding the role of this cytokine in tumor biology, it is diverse and opposite. This is mainly due to different isoforms that are located in tumor tissue. Expression of this cytokine in tumor tissue is, in most cases, higher than that in peritumoral or normal tissue and has a prognostic significance; the higher expression usually is strongly correlated with worse prognosis and more progressive form of disease [24, 25]. Some literature data showed antitumorigenic effect of this cytokine [26]. Its role in tumor angiogenesis is still controversial and less defined.

There are almost no data about the role and expression pattern of this cytokine in different histological forms of gastric cancer and intestinal and diffuse type of gastric cancer. The aim of this study is to reveal some data about expression and possible role in gastric carcinogenesis, especially in these two tumor types: diffuse and intestinal gastric cancer.

## 2. Material and Methods

### 2.1. Ethic Approvals

The study was conducted at the Center for Abdominal Surgery, Center for Pathology, Clinical Center of Kragujevac, and Center for Molecular Medicine and Stem Cell Research, Faculty of Medical Sciences, University of Kragujevac, Serbia. All patients gave their informed consent. Ethical approvals were obtained from relevant Ethics Committees of the Clinical Center of Kragujevac, Kragujevac, Serbia, and Faculty of Medical Sciences, University of Kragujevac, Serbia (number 01-11478). All research procedures were made according to the *Principles of Good Clinical Practice* and the Declaration of Helsinki.

### 2.2. Patients

The study included 70 patients with gastric cancer. The diagnosis of gastric cancer was based on gastroscopic and histopathological criteria. The study excludes patients with no well-defined pathology, inadequate clinical document, or with previously diagnosed gastric cancer who were treated with radiation and chemotherapy. In the present study, we analyzed clinical data about age, gender, pathologic reports (nuclear grade and well/moderate/poor differentiation), and clinical stage by TNM (tumor, nodes, and metastasis) of patients with gastric cancer. Well-differentiated and moderately differentiated tumors (well/moderate) were defined as low-grade lesions, whereas poorly differentiated tumors (poor) were defined as high-grade lesions according to the WHO guidelines [27]. Grading was based on the evaluation of the worst area, excluding areas of focal dedifferentiation present at the invasive margin of the tumor [28]. Poorly differentiated tumors have repeatedly been shown to behave more aggressively than well-/moderately differentiated carcinomas in multivariate analysis [28]. The classification of nuclear grade of tumor tissue (I + II and III + IV) was based on the evaluation of the size and shape of the nucleus in tumor cells and the percentage of tumor cells that are in the process of dividing or growing [29].

### 2.3. Immunohistochemical Staining of VEGF, IL-32, IL-17, and CD31

Paraffin-embedded samples were consecutively cut to a thickness of 4–5 *μ*m. Each section was deparaffinized and rehydrated with graded ethanol. Antigen retrieval was performed by microwave heating for 20 minutes in 10 mM sodium citrate buffer (pH 6.0). Activity of endogenous peroxidase was blocked with a 3% hydrogen peroxide solution for 10 min at room temperature. After washing with PBS, slides were incubated with mono/polyclonal antibodies against VEGF (ab16883, Abcam, Cambridge, UK, at a 1 : 200 dilution), IL-32 (ab37158, Abcam, Cambridge, UK, at 10 *μ*g/ml), IL-17 (ab79056, Abcam, Cambridge, UK, at a 1 : 100 dilution), and CD31 (ab79056, Abcam, Cambridge, UK, at a 1 : 200 dilution) for 60 min in a humid chamber, respectively. Sections were washed in PBS three times and then incubated with anti-rabbit/mouse secondary antibody, respectively, for 15 min at room temperature. Immunostaining was performed using the Envision system with diaminobenzidine (DakoCytomation, Glostrup, Denmark). Finally, the signal was developed with 3,3-diaminobenzidine tetrahydrochloride (DAB), and all of the slides were counterstained with hematoxylin. Negative controls were treated in the same way with the primary antibodies omitted. Positive controls consisted of tissue known to contain the protein of interest [30]. An Olympus microscope (BX50 model) equipped with a digital camera was used to prepare microphotographs with magnifications of 200x or 400x.

### 2.4. IHC Scoring

Two independent pathologists investigated all tissue specimens. The tissue samples were analyzed using semiquantitative modified scoring system, according to the percentage of tumor tissue stained with IL-32 and intensity of staining [25, 31]. The IHC score was calculated by addition of the percentage of positively stained cells to the staining intensity. The percentage of positive cells ranged between 0 and 3, i.e., 0, if less than 10% of tumor cells were stained; 1, if 10–25% of tumor cells were stained; 2, if 25–50% were positive; and 3, if >50% were positive. The staining intensity was scored as 0 for negative, 1 for weak, 2 for moderate, and 3 for strong intensity. The IHC score was ranged between 0 and 6.

VEGF scoring was calculated according to the presence, intensity, and percent of positive cells, as previously described [30, 31]. Brown or brown-yellow staining in the cell membrane or cytoplasm was considered as positive. The negative controls were unstained. The number of positive cells in 500 tumor cells was counted within 3 randomly selected high-power fields (×400). Four grades were defined according to the percentage of positively stained cells: 0, no immunopositive cells; 1, <25% immunopositive cells; 2, 25–50% immunopositive cells; 3, >50% immunopositive cells. Four grades were defined according to color-staining intensity: 0, no color; 1, weak, pale yellow; 2, medium brown; 3, strong, dark brown.

Two independent pathologists considered CD-31-positive single endothelial cells or CD-31-positive clusters of endothelial cells as a microvessel. At first, slides were examined at an original magnification of 40x. Three “hot spots” (areas with the highest microvessel density) from each slide were identified and these are as were photographed by a digital camera at an original magnification of 200x. The area of this histological field was 0.704 *μ*m. MVD (microvessel/HPF) and the number of microvessels evaluated according to Weidner et al. (1991). MVD of the specimen was estimated as a mean of MVD in three histological fields.

Expression of IL-17 was localized in the cytoplasm of mononuclear cells. Light microscopic analysis was performed by manually counting positively stained cells in 3 separate areas of intratumoral regions under 400x high-power magnifications [32].

### 2.5. Statistical Analysis

The data were analyzed using the commercially available SPSS 20.0 software. The results were reported as mean and standard error of mean (SEM). Results were analyzed using the Student's *t*-test for independent samples if the data had normal distribution or Mann–Whitney *U* test for data without normal distribution. Spearman's correlation assessed the possible relationship between the IL-32 expression and histological form of gastric cancer. Strength of correlation was defined as negative or positive weak (−0.3 to −0.1 or 0.1 to 0.3), moderate (−0.5 to −0.3 or 0.3 to 0.5), or strong (−1.0 to −0.5 or 1.0 to 0.5). Statistical significance was set at *p* < 0.05.

## 3. Results

Seventy patients with gastric cancer were enrolled in this study. Clinical and pathologic characteristics of these patients are presented in Table 1. Patients with gastric cancer were divided in two groups on the basis of type of tumor: diffuse form and intestinal form of gastric cancer. Significant difference was observed in gender distribution (*p* = 0.025). Histopathological analysis confirmed that 12 female patients had diagnosed diffuse type of gastric cancer while 41 male patients had intestinal form of gastric cancer (41 males and 8 females). Moreover, significant difference was observed in age between patients with diffuse (mean age 65.2 ± 2.72) and intestinal type of gastric cancer (mean age 75.07 ± 1.13). Patients with diagnosed intestinal form of gastric cancer were significantly greater (*p* = 0.005) in comparison to patients with diffuse form of tumor (Table 1).

### 3.1. More Severe and Aggressive Disease Associated to Diffuse Form of Tumor

Patients with different forms of gastric cancer were divided into two categories on the basis of TNM stage of disease: I + II and III + IV. As shown in Table 1, patients with diffuse form of tumor appear to have an advanced TNM stage of disease (TNM stage III + IV) (*p* = 0.045), while patients with intestinal form of gastric cancer mostly had localized tumor (TNM stage I + II).

Patients with diffuse form of gastric cancer appeared to have higher nuclear grade (*p* = 0.001), while patients with intestinal form of gastric cancer mostly had lower nuclear grade (Table 1).

Further, we analyzed patients with different forms of gastric cancer, according to histological differentiation rate: well/moderate and poor. Majority of patients with diffuse form of cancer had poor tumor tissue differentiation (*p* = 0.001), while patients with intestinal form of gastric cancer had mostly better tumor tissue differentiation (Table 1). According to results from Table 1, TNM classification and nuclear and histological grade suggested that patients with diffuse type of gastric cancer have more severe form of disease compared to patients with intestinal form of tumor.

### 3.2. Lower Expression of IL-32 Associated to Diffuse Form of Gastric Cancer

**Figure 1:**
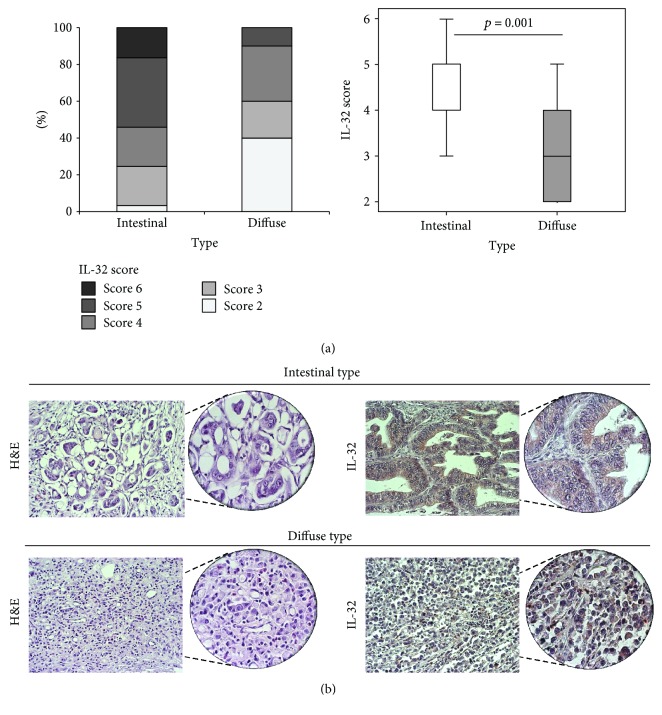
IL-32 score in patients with intestinal and diffuse form of gastric cancer. (a) Patients with diffuse type of cancer had IL-32 score 4 or less, while patients with intestinal type had IL-32 score 4 or higher. Significantly lower IL-32 score in patients with diffuse type in comparison to patients with intestinal type of gastric cancer (*p* = 0.001). *p* values were assessed by Student's unpaired *t*-test. (b). H&E staining of representative tumor tissue of intestinal and diffuse type of gastric cancer. Representative IL-32 staining in patients with intestinal and diffuse type of gastric cancer (200 and 400x magnification).

The results have shown that majority of patients with diffuse type of gastric cancer had score 4 or less, while most of the patients with intestinal form of tumor had score 4 or higher (*p* = 0.001; Figure 1(a), right panel). Within patients with diffuse type of gastric cancer, IL-32 score 2 was recorded for 40% of patients, while IL-32 score 2 was recorded for 3% of patients with intestinal type of gastric cancer (Figure 1(a), left panel). Moreover, Spearman's correlation test revealed that higher expression of IL-32 negatively correlates with more severe diffuse form of gastric cancer (*r* = −0.367; *p* = 0.002).

### 3.3. Lower Microvascular Density and IL-17 Expression in Diffuse Form of Gastric Cancer

**Figure 2:**
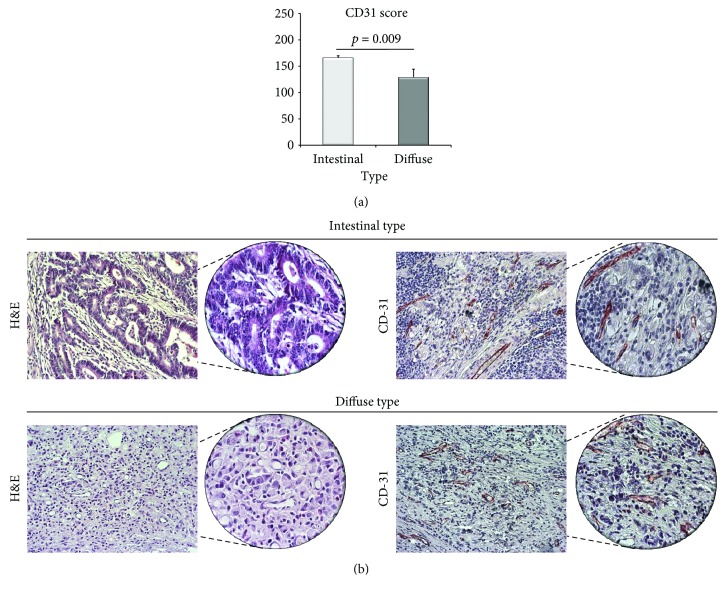
Microvascular density of intestinal and diffuse form of gastric cancer. (a) MVD was significantly lower in patients with diffuse form compared to patients with intestinal form of gastric cancer (*p* = 0.009). *p* values were assessed by Mann–Whitney rank sum test. (b) H&E staining of representative tumor tissue of intestinal and diffuse type of gastric cancer. Representative sections demonstrate MVD in tumor tissue of patients with intestinal and diffuse type of gastric cancer (200 and 400x magnification).

Immunohistochemistry results have shown that patients with diffuse form of tumor have significantly lower MVD in comparison to patients with intestinal type of gastric cancer (*p* = 0.009), suggesting on dramatically less level of angiogenesis in diffuse form of gastric cancer (Figure 2(a)).

**Figure 3:**
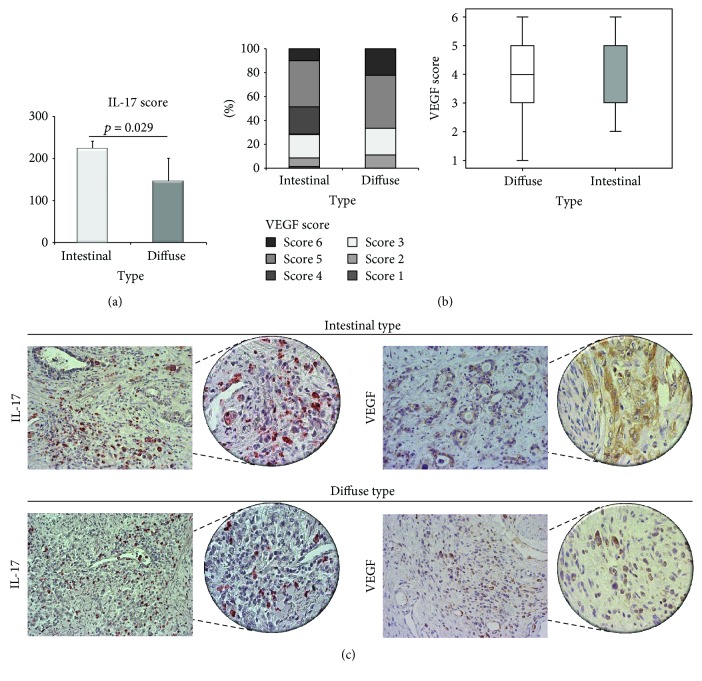
Immunohistochemical analysis of IL-17 and VEGF in patients with intestinal and diffuse form of gastric cancer. (a) Significantly lower IL-17 score in patients with diffuse type in comparison to patients with intestinal type of gastric cancer (*p* = 0.029). (b) No statistical significance in VEGF score between patients with diffuse form and intestinal form of gastric cancer (*p* > 0.05). *p* values were assessed by Mann–Whitney rank sum test. (c) Representative IL-17 and VEGF staining in tumor tissue of patients with intestinal and diffuse type of gastric cancer (200 and 400x magnification).

There was no statistical difference in VEGF expression between patients with diffuse and intestinal type of gastric cancer (Figure 3(b)). Analyses of IL-17 expression have revealed that patients with diffuse form of gastric cancer had significantly lower expression of this cytokine in comparison to patients with intestinal tumor form (*p* = 0.029; Figure 3(a)).

## 4. Discussion

Gastric cancer is one of the most frequently diagnosed malignancies and the second cause of cancer-related death in population [2]. According to Lauren's classification as well as the World Health Organization, there are two major histological entities of gastric cancer: intestinal and diffuse type [33, 34]. *Helicobacter pylori* infection; *Helicobacter pylori*-associated chronic gastritis, atrophy, and intestinal metaplasia; lifestyle; and diet are the main risk factors for the development of intestinal type of gastric cancer [35, 36]. On the contrary, diffuse type of this tumor is more frequently linked to with genetic mutations [2]. Intestinal type of gastric cancer consists of tubular or glandular metaplastic cell formations, while poorly differentiated diffuse form of tumor is usually formed of cells without gland formation, with the presence of signet ring cells and mucin [37, 38]. Our results have shown that diffuse form of cancer dominated in younger female patients while intestinal form of gastric cancer was more frequent in elder male patients (Table 1). Moreover, TNM classification, nuclear grade, and histological score clearly suggested that diffuse form of cancer is more severe than intestinal form (Table 1). These results are in line with previous reports claiming that intestinal gastric cancer most commonly occurs in elderly male patients and exhibits a longer course and better prognosis, while diffuse form is often associated with younger age, predominantly in younger women with worse prognosis [9, 38].

In order to investigate potential biological role of IL-32 in obvious difference in severeness of diffuse form of gastric cancer in comparison to intestinal form, we have analyzed the expression of IL-32 in tumor tissue. Our results revealed higher IL-32 expression in patients with diffuse type of gastric cancer in comparison to intestinal form of tumor. It has been reported that systemic concentration of IL-32 is significantly increased in patients with gastric cancer in comparison to healthy control [24]. Ishigami et al. have shown that tumor depth and lymph node metastases as well as lymphatic and venous invasion developed more frequently in IL-32-positive gastric cancer [39]. Earlier study also confirmed that expression of IL-32 in patients with gastric cancer positively correlated with poor prognosis. Moreover, IL-32 by promoting production of MMP2, MMP9, IL-8, and VEGF facilitates invasion as well as migration of tumor cells [40]. Interestingly, all the data refer to intestinal type of gastric cancer.

The degree of microvascular density in tumor is nowadays assessed by CD31 protein expression. Platelet/endothelial cell adhesion molecule-1 (PECAM-1) or CD31 is a multifunctional molecule involved in different processes like platelet biology, signal transduction, transendothelial migration of leukocytes, and inflammation as well as endothelial cell biology [41]. Moreover, CD31 plays an important role in tumor biology in few ways. It is one of the most abundant junctions set deep between endothelial cells, thus supporting the integrity of endothelial membrane and regulating leukocyte migration and vascular permeability [41, 42]. Our results have revealed that diffuse form of gastric cancer had significantly lower MVD in comparison to intestinal form of tumor. Previous reports have suggested that intestinal form of gastric cancer spreads predominantly in the liver via direct hematogenous way, while diffuse gastric cancer is more invasive and gives metastatic lesions directly in peritoneal cavity. The reason for this different way of spreading tumors is the fact that intestinal form is more dependent on angiogenesis in comparison to diffuse form of tumor [43]. In line with the previous findings are our results suggesting that decreased MVD reflects less degree of angiogenesis in diffuse form in comparison to intestinal form of gastric cancer.

As the reason for this significant difference in microvascular density can be the presence or absence of different pro-/antiangiogenic markers, in the continuation of our research, we have focused on analyzing expression of these factors in diffuse and intestinal form of gastric cancer. First, we have analyzed expression of vascular endothelial growth factor (VEGF), which is one of the most potent proangiogenic factors. VEGF is an endothelial cell-specific mitogen which is important for endothelial cell survival, proliferation, and migration [44, 45]. The main sources of this factor are various cell types such as tumor cells, macrophages, or platelets [46]. Abundant expression of VEGF has an important role in the pathogenesis of cancer, proliferation of tumor cells, and the development of metastatic lesions [47, 48]. However, we have not found significant difference between expression of VEGF in diffuse and intestinal form of gastric cancer. This result suggests that difference in microvascular density between diffuse and intestinal type of gastric cancer is not caused by VEGF.

IL-17 is a cytokine produced mainly by Th17 cells, although other types of cells such as *γδ* T lymphocytes and type 3 innate lymphoid cells can also be important sources of this cytokine [49]. Previous reports suggested that IL-17 is abundantly expressed in different forms of tumors and that its concentration positively correlates with VEGF expression in tumors [50]. Moreover, Iida et al. have shown that patients whose infiltrates in gastric cancers had increased number of Th17 cells with increased expression of IL-17 and IL-23 mRNA had more invasive form of tumors [32]. Our analyses of IL-17 expression in gastric cancer have showed that patients with diffuse form of tumor had significantly lower expression of this cytokine compared to patients with intestinal form of the tumor (Figure 3(a)). This result is in line with previous studies suggesting that IL-17 has an important role as a proangiogenic factor [51]. Moreover, significant lower expression of IL-17 and no detectable difference in VEGF expression suggest that diminished IL-17 may cause reduce angiogenesis and subsequent milder microvascular density in diffuse type of gastric cancer.

**Figure 4:**
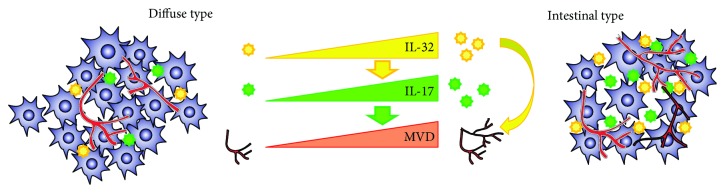
Schematic diagram describing mechanism responsible for IL-32-mediated suppression of angiogenesis in diffuse type of gastric cancer. IL-32 directly and indirectly, through the suppression of IL-17, reduces angiogenesis and subsequent microvascular density, which in turn attenuates hematogenous metastasis of diffuse type of gastric cancer.

According to the presented data, it appears that decreased expression of IL-32 may inhibit production of proinflammatory and proangiogenic factor IL-17 and thus suppresses formation of new blood vessels which in turn results in diminished hematogenous metastatic potential of diffuse form of cancer (Figure 4).

## 5. Conclusions

In summary, decreased local presence of IL-32, reflected through a lower expression, in diffuse type of gastric cancer patients, with a higher nuclear grade, poor tumor tissue differentiation, and advanced TNM stage of disease, may be considered as a sign of the tumor's malignant progression and, consequently, of a poor prognosis for patients. This finding throws a new light on the role of IL-32 in biology of diffuse form of gastric cancer.

## Figures and Tables

**Table 1:** Baseline characteristics of patients with intestinal and diffuse type of GC.

	Gastric cancer		*p*
	Intestinal type (*n* = 50)	Diffuse type (*n* = 20)	
Gender (male/female)	41/9	8/12	0.025
Age (mean (range))	75.07 (54–92)	65.20 (55–79)	0.005
TNM classification (I and II/III and IV)	30/20	6/14	0.045
Nuclear grade (I/II/III)	4/35/11	0/0/20	0.001
Histological differentiation rate (well/moderate/poor)	11/26/13	0/0/20	0.001
Blood vessel invasion (absent/present)	37/13	8/12	0.011

## Data Availability

All data used to support the findings of this study are included within the article.
